# Personality Traits in Fibromyalgia (FM): Does FM Personality Exists? A Systematic Review

**DOI:** 10.2174/1745017901814010263

**Published:** 2018-10-31

**Authors:** Ciro Conversano, Laura Marchi, Rebecca Ciacchini, Claudia Carmassi, Bastianina Contena, Laura Maria Bazzichi, Angelo Gemignani

**Affiliations:** 1 Department of Surgical, Medical and Molecular Pathology, Critical and Care Medicine, University of Pisa, Pisa, Italy; 2 Department of Clinical and Experimental Medicine, Rheumatologic Clinic, University of Pisa, *via* Roma 67, 56100 Pisa, Italy; 3 Department of Clinical and Experimental Medicine, University of Pisa, *via* Roma 67, 56100 Pisa, Italy; 4 Department of Health Science, University of Florence, Florence, Italy

**Personality Traits in Fibromyalgia (FM): Does FM Personality Exists? A Systematic Review**

Clinical Practice & Epidemiology in Mental Health, **2018**, 14: 223-232

**Correction**

The corrections are provided and replaced online which is mentioned as under:

**Original:**

 The name of coauthor was Ciacchini Rebecca

**Corrected:**

The name of coauthor has been revised as Rebecca Ciacchini

